# Retraction Note: Genetically modified macrophages accomplish targeted gene delivery to the inflamed brain in transgenic Parkin Q311X(A) mice: importance of administration routes

**DOI:** 10.1038/s41598-025-27452-5

**Published:** 2025-11-25

**Authors:** Matthew J. Haney, Yuling Zhao, James Fay, Hwang Duhyeong, Mengzhe Wang, Hui Wang, Zibo Li, Yueh Z. Lee, Mohan K. Karuppan, Nazira El-Hage, Alexander V. Kabanov, Elena V. Batrakova

**Affiliations:** 1 Center for Nanotechnology in Drug Delivery, University of North Carolina at Chapel Hill, Chapel Hill, NC USA; 2 UNC Eshelman School of Pharmacy, University of North Carolina at Chapel Hill, Chapel Hill, NC 27599-7362 USA; 3 Biomedical Research Imaging Center, University of North Carolina at Chapel Hill, Chapel Hill, NC USA; 4 Department of Radiology, University of North Carolina at Chapel Hill, Chapel Hill, NC USA; 5 Department of Immunology, Herbert Wertheim College of Medicine, Florida International University, Miami, FL 33199 USA

Retraction of: *Scientific Reports* 10.1038/s41598-020-68874-7, published online 16 July 2020

The Editors have retracted this Article at the request of the University of North Carolina at Chapel Hill.

An investigation conducted by the University regarding the Article concluded that results reported in Figures 1 and 2 were falsified. The data originated from experiments unrelated to those described in the Figure captions, and the images were found to originate from another article [1]. The Editors therefore no longer have confidence in the research presented in this work.

Yuling Zhao, Yueh Z. Lee, and Alexander V. Kabanov agree with the retraction and its wording. Elena V. Batrakova disagrees with the retraction. Matthew J. Haney, James Fay, Hwang Duhyeong, Mengzhe Wang, Hui Wang, Zibo Li, Mohan K. Karuppan, and Nazira El-Hage did not respond to the correspondence from the Editors about this retraction.
